# Texture modified diet in German nursing homes: availability, best practices and association with nursing home characteristics

**DOI:** 10.1186/s12877-019-1286-9

**Published:** 2019-10-23

**Authors:** Carina Burger, Eva Kiesswetter, Rowena Alber, Ulrike Pfannes, Ulrike Arens-Azevedo, Dorothee Volkert

**Affiliations:** 1 Institute for Biomedicine of Aging, Friedrich-Alexander-Universität Erlangen-Nürnberg, Kobergerstraße 60, 90408 Nuremberg, Germany; 2 Faculty Life Sciences, University of Applied Sciences Hamburg, Hamburg, Germany

**Keywords:** Nursing home, Texture modified diet, Swallowing problems, Chewing problems, Nutritional care

## Abstract

**Background:**

For nursing home (NH) residents with swallowing or chewing problems, appealing texture-modified-diets (TMD) need to be available in order to support adequate nutrition. The aim of this study was to describe the availability of TMD and best practices for TMD in German NHs and to identify related NH characteristics.

**Methods:**

Information on NH characteristics, available texture-modified (TM)-levels (soft, “minced & moist”, pureed) and implemented best practices for TMD (derived from menu plan, separately visible components, re-shaped components, considering individual capabilities of the resident) was collected in a survey in German NHs. The number of TM-levels as well as the number of best practices for TMD were tested for their association with 4 structural, 16 operational and 3 resident-related NH characteristics.

**Results:**

The response rate was 7.2% (*n* = 590) and 563 NHs were included. The vast majority of NHs (95.2%) reported offering “minced & moist” texture and 84.2% preparing separately visible meal components. Several operational characteristics were more frequently (*p* < 0.05) reported from NHs offering three TM-levels (27.7%) or four best practices for TMD (13.0%) compared to NHs offering one TM-level (28.4%) or one best practice for TMD (20.1%): special diets and delivery forms (e.g. fingerfood 71.2% vs 38.8%; 80.8% vs. 44.3%), written recipes (69.9% vs. 53.1%; 68.5% vs. 53.9%), a dietetic counseling service (85.9% vs. 66.3%; 89.0% vs. 65.2%), a quality circle for nutritional care (66.7% vs. 43.8%; 71.2% vs. 50.4%), regular staff training (89.7% vs. 73.1%; 95.9% vs. 74.8%) and process instructions (73.7% vs. 53.1%; 75.3% vs. 47.8%). No associations were found regarding structural and resident-related NH characteristics, except a higher percentage of residents receiving TMD in NHs with three compared to one TM-level (median 16.3% vs. 13.2%, *p* = 0.037).

**Conclusion:**

All participating NHs offer some form of TMD, but only a small number offers a selection of TMD and pays adequate attention to its preparation. Operational NH characteristics – which might reflect a general nutritional awareness of the NH – seem to be pivotal for provision of TMD, whereas neither structural nor resident-related characteristics seem to play a role in this regard.

## Background

Nursing homes face the challenge of having to ensure adequate nutritional care, considering differing requirements and wishes of their residents at the same time. The provision of food for all meals of the day including special diets and delivery forms is part of this challenge. As many residents are not able to eat food of regular consistency due to chewing or swallowing problems, texture-modified diets are also required.

The prevalence of chewing problems increases with age and is higher in nursing home residents than in community-dwelling older people [1]. The prevalence of swallowing problems increases with age as well as these problems are concomitant of several age-related diseases like dementia, stroke or Parkinson’s disease [2, 3]. High prevalence numbers of swallowing (7–68%) and chewing (11–57%) problems in NHs are described in a review of 17 studies, with 54 to 6832 participants reporting nutritional problems in nursing home residents [4]. Another more recent review reported prevalence rates for swallowing problems ranging between 7 and 40%, considering eight studies with 40 to 136,794 participants in long-term care [5].

Texture-modified diet (TMD) is rarely a diet of choice, but necessary for individuals with swallowing or chewing problems to enable them to safely eat orally in spite of a high risk of aspiration [6]. Texture modification often results in less appealing, less tasteful and nutritionally diluted foods that are poorly accepted [7–9], characteristics which in turn may contribute to reduced quality of life, malnutrition and dehydration [10]. The proportion of nursing home residents receiving TMD ranges between 26 and 67%, according to a recent review on use of texture-modified food and fluid intake in dementia and residential care facilities, considering four studies from Canada, USA and Australia [9].

The ability to chew and swallow food varies among residents depending on the type and severity of their disorders. Therefore, different degrees of texture modification are required to ensure a safe swallowing process as well as to maintain and improve the ability to eat. In a framework on texture-modified foods and thickened fluids recently published by the International Dysphagia Diet Standardization Initiative (IDDSI), four adaption levels for food (soft & bite sized, minced & moist, pureed, liquidized) are recommended with the aim to standardize terminology and definitions for texture modified foods and thickened fluids internationally [11].

The German Nutrition Society (DGE) has published national recommendations for nutrition in nursing homes with specific recommendations for TMD [12]. These recommendations include the advice to offer several TM-levels (e.g. soft, partly minced, minced and moist, pureed) and to consider the following best practices for TMD: 1. Texture modified meals should be based on the same ingredients as regular meals (“derived from menu plan”), 2. Meal components should be separately visible, 3. Meal components should be re-shaped. 4. The level of TMD should be based on the individual capabilities of the resident [12]. Similar recommendations are made in the “Best Practice Food and Nutritional Manual for Aged Care Homes” by the Central Coast Local Health District in Australia [13].

Presently, it is not clear whether the DGE recommendations for TMD are known and implemented in German NHs. Within the framework of a national survey on catering and nutritional care in German NHs, we thus examined the availability of TMD and best practices for TMD. We further wanted to identify NH characteristics related to the provision of TMD.

## Methods

### Study design

This nationwide cross-sectional survey was part of a larger project on behalf of the German Nutrition Society [14]. The required contact information of 10,589 (97% of all German) NHs was provided by the scientific institute of a large German health insurance company (AOK, Allgemeine Ortskrankenkasse). In order to achieve equal participation throughout Germany, five regions with similar numbers of NHs were predefined according to federal states [15]. In March 2014, a written questionnaire was sent to the management of a random sample of 5000 NHs (1000 for each region). For the participation, cooperation with the head of the nursing service and the kitchen manager was recommended. The questionnaire could be completed online or sent back as hardcopy by postal mail. As the return rate was below 5% after 4 weeks, all addressed NHs with available e-mail information (4156 NHs) received a reminder for the survey. In addition, the sample was enlarged by all NHs in the AOK list with available e-mail information which were not addressed before (3172 NHs). In total, 8172 NHs received an invitation to the survey.

### Questionnaire

The questionnaire was developed by the authors and pilot-tested in ten NHs. The final questionnaire consisted of 48 questions with mostly predefined answer categories (Additional file 1). For this analysis, two questions regarding TMD and 15 questions covering structural, operational and resident-related NH characteristics were used. An item-selection was made for four multiple-choice questions regarding operational NH characteristics with a comprehensive item set, choosing the most relevant items for this context. Additionally, a question on the knowledge of the national recommendations for nutrition in NHs (“DGE quality standard” known (yes/no)) was posed.

#### Texture modified diet

1. The availability of four levels of TMD was examined with the help of a multiple-choice question that included the following answer options: “soft (not pureed)”/“minced and moist” (finely minced)/“pureed (strained through a sieve)”/“smooth food”). Short explanations were provided to allow correct assignment.
2. The existence of best practices for TMD was checked with a multiple-choice question with the following items: components are derived from menu plan/components are separately visible/components are re-shaped/individual capabilities of the residents are considered.

#### Structural NH characteristics

Relevant structural data, namely the respective city size (≤/> 20,000 inhabitants), the type of partnership (non-profit/for-profit), and the place of production of hot meals (in-house/external) was collected with the help of closed questions. The number of beds for inpatient care was inquired with the help of an open question, and NHs were grouped into small (1–50 beds), medium (51–100 beds) and large (> 100 beds) institutions.

#### Operational NH characteristics

1. With regard to food provision and menu planning, the availability of special diets and delivery forms (vegetarian/Muslim/energy-dense/finger food; as part of a 14-item multiple-choice question), the availability of written recipes (yes, for all dishes/yes, for most dishes/yes, for some dishes/no), and the calculation of energy and nutrient content for at least one menu line (yes/no/don’t know) was examined.
2. Nutritional care was queried by the following items: frequency of malnutrition screening (once at admission/1–2 times per year/4–6 times per year/about once a month/never), availability of dietetic counseling service (as part of a six-item multiple-choice question regarding service and assistance available) and routine assessment of the personal nutrition history of all residents (yes/no).
3. Management and quality assurance regarding nutrition were assessed with the help of the following questions: availability of a dietician (yes/no), implemented quality assurance aspects (regular staff training and regular surveys on resident satisfaction as part of a seven item multiple-choice question) and implemented regulations for interface management between kitchen, nursing care and housekeeping (interdisciplinary nutrition team, periodic quality circle, process instructions and interface descriptions as part of a seven-item multiple-choice question).

The two non-polar questions “availability of recipes” and “frequency of malnutrition screening” were dichotomized according to national recommendations [12].

#### Resident-related NH characteristics

The number of residents with swallowing disorders, the number of residents with chewing disorders and the number of residents receiving texture modified food and/or fluid were examined with the help of open questions. The percentage of residents with each characteristic was calculated by dividing these numbers by the number of beds for inpatient care.

### Data analysis

All results are presented for the total group and for subgroups according to the number of available texture modified (TM)-levels and best practices for TMD. The number of available TM-levels was calculated excluding “Smoothfood”, as this is usually not provided as a complete diet but only offered additionally, e.g. for residents relying on a feeding tube. The number of best practices for TMD was also calculated. The data are presented as relative frequencies (TMD, structural and operational NH characteristics), and mean ± standard deviation, median, interquartile range (IQR) (resident-related NH characteristics).

Differences between groups were tested using Chi^2^ tests. A post-hoc analysis was conducted with z-tests and Bonferroni corrections to specify differences. Kruskal-Wallis tests were used to identify differences in resident-related NH characteristics as a function of the number of TM-levels and the number of considered best practices for TMD. Pairwise comparisons with Bonferroni corrections were used to specify differences. Missing values were not included in statistical tests. Statistical significance was set at a *p*-value of < 0.05.

Statistical analyses were performed with SPSS 24.0.

## Results

In total, 590 NHs completed and returned the questionnaire (response rate 7.2%). Ten questionnaires were excluded due to missing data on TMD, and 17 questionnaires were excluded as more than 15% of other values of interest were missing. The analysis included 563 NHs with 10 to 390 (89.5 ± 50.2, median 81) beds, of which 57.9% were non-profit institutions. Furthermore, 27.9% of the NHs were located in the region South, 16.5% in the region East, 19.7% in the region North, 16.0% in the region Central Germany and 19.9% in the region North Rhine-Westphalia.

### Texture modified diet (TMD)

The availability of TMD is presented in Table 1 for the total group and stratified according to the number of TM-levels and the number of best practices for TMD. Almost all NHs (95.2%) stated to offer “minced & moist” texture. Nearly half of the NHs (43.9%) had two different TM-levels available, and about one quarter offered one (28.4%) or three (27.7%) TM-levels. All combinations of the three TM-levels were found, with soft and “minced and moist” texture being the most common (29.8%) (Additional file 2). “Smooth food” was only offered in a sixth of all NHs (17.1%). The proportion of NHs offering “Smoothfood” increased with the number of available TM-levels (*p* < 0.001) as well as with the number of best practices for TMD (*p* < 0.001) (Table 1).

**Table 1:** Texture modified diet in German NHs (overall and according to number of TM-levels and best practices for TMD) [%]

	Total (*n* = 563)	Number of TM-levels				Number of best practices for TMD				
		1 (*n* = 160)	2 (*n* = 247)	3 (*n* = 156)	p	1 (*n* = 115)	2 (*n* = 166)	3 (*n* = 209)	4 (*n* = 73)	p
TM-levels										
Soft	59.9	2.5	71.7	100.0		39.1^a^	51.2^a^	70.8^b^	80.8^b^	< 0.001
“Minced and moist”	95.2	88.8	96.4	100.0		91.3	96.4	95.7	97.3	0.163
Pureed	44.2	8.8	32.0	100.0		37.4^a^	39.8^a^	43.5^a^	67.1^b^	< 0.001
“Smoothfood”	17.1	9.4^a^	13.8^a^	30.1^b^	< 0.001	5.2^a^	16.3^b^	14.8^a,b^	43.8^c^	< 0.001
Best practices for TMD										
Components derived from menu plan	68.0	62.5^a^	65.2^a^	78.2^b^	0.005	31.3	53.6	88.5	100.0	
Components separately visible	84.2	78.1^a^	84.2^a,b^	90.4^b^	0.012	51.3	83.1	97.6	100.0	
Components re-shaped	27.9	21.3^a^	21.9^a^	44.2^b^	< 0.001	4.3	13.3	27.3	100.0	
Individual capabilities considered	63.1	46.3^a^	67.2^b^	73.7^b^	< 0.001	15.7	50.0	86.6	100.0	

Differences by number of TM-levels and number of best practices for TMD were tested with Chi^2^-test and post-hoc z-test, respectively. Each subscript letter denotes a subset of “number of TM-levels” or “number of best practices for TMD” categories whose column proportions do not differ significantly from each other (*p* < 0.05)

One in five NHs (20.4%) implemented only one of the four best practices for TMD. Two best practices for TMD were considered by 29.5%, three by 37.1% and all four by 13.0% of the NHs. All combinations of the four best practices for TMD were found (Additional file 3). Most commonly implemented was the best practice “components separately visible”, namely by 84.2% of the NHs. “Re-shaped components”, considered by 27.9% of the NHs, was the least common best practice (Table 1).

NHs having three TM-levels available considered each of the best practices for TMD more frequently (all *p* < 0.02) than NHs with only one TM-level. Furthermore, NHs considering four best practices for TMD offered soft texture (*p* < 0.001) and pureed texture (*p* < 0.001) more frequently than NHs considering only one or two best practices for TMD (Table 1). Overall, 7.6% of the NHs stated to offer all of the three TM-levels and all of the four best practices for TMD, whereas 9.5% of the NHs offered only one TM-level and implemented only one best practice for TMD.

The national recommendations for nutrition in NHs are known by 69.8% of the NHs regardless of the available number of TM-levels or the number of best practices for TMD (Additional file 4).

### Structural NH characteristics and TMD

None of the structural NH characteristics were associated with the number of available TM-levels or the number of best practices for TMD (Table 2). Solely the production of hot meals in-house tended to be more common in NHs with three or four best practices for TMD compared to NHs with only one or two (*p* = 0.061) (Table 2).

**Table 2:** Structural characteristics of German NHs (overall and according to number of TM-levels and best practices for TMD) [%]

		Number of TM-levels				Number of best practices for TMD				
	Total (*n* = 563)	1 (*n* = 160)	2 (*n* = 247)	3 (*n* = 156)	p	1 (*n* = 115)	2 (*n* = 166)	3 (*n* = 209)	4 (*n* = 73)	p
Institutional characteristics										
City size										
≤20.000 inhabitants	51.2	52.5	52.6	47.4	0.457	50.4	56.0	50.2	43.8	0.294
>20.000 inhabitants	46.0	44.4	44.1	50.6		46.1	41.0	46.9	54.8	
Partnership										
Non-profit	57.9	56.9	57.9	59.0	0.952	57.4	63.9	54.1	56.2	0.305
For-profit	40.9	41.9	40.1	41.0		40.0	35.5	45.0	42.5	
NH size										
1–50 beds	17.4	13.8	21.9	14.1	0.180	18.3	22.3	13.9	15.1	0.198
51–100 beds	53.1	56.3	49.8	55.1		56.5	47.6	57.9	46.6	
> 100 beds	28.4	28.8	27.1	30.1		25.2	28.9	27.3	35.6	
Foodservice characteristics										
Hot meal production										
In-house	82.2	81.9	81.8	83.3	0.943	79.1	77.7	85.6	87.7	0.061
external	16.2	16.3	16.6	15.4		20.0	20.5	12.9	9.6	

The percentage of missing values is the difference to 100%, respectively

Differences by number of TM-levels and number of best practices for TMD were tested with Chi^2^-test and post-hoc z-test, respectively. Each subscript letter denotes a subset of “number of TM-levels” or “number of best practices for TMD” categories whose column proportions do not differ significantly from each other (*p* < 0.05)

### Operational NH characteristics and TMD

#### Food provision and menu planning

The availability of special diets and delivery forms increased with an increasing number of available TM-levels and best practices for TMD (Table 3). Recipes for most/all dishes were more frequently available in NHs offering three than in NHs offering only one or two TM-levels (*p* = 0.002). They were also more frequently available in NHs using three or four best practices for TMD than in NHs considering only two (*p* = 0.007). The energy and nutrient content of at least one menu line was calculated by two thirds of the NHs without differences in both TMD aspects (Table 3).

**Table 3:** Operational characteristics of German NHs (overall and according to number of TM-levels and best practices for TMD) [%]

		Number of TM-levels				Number of best practices for TMD				
	Total (*n* = 563)	1 (*n* = 160)	2 (*n* = 247)	3 (*n* = 156)	p	1 (*n* = 115)	2 (*n* = 166)	3 (*n* = 209)	4 (*n* = 73)	p
Food Provision and Menu planning										
Special diets and delivery forms										
Vegetarian diet	75.8	70.0^a^	73.7^a^	85.3^b^	0.004	70.4^a^	72.9^a,b^	77.0^a,b^	87.7^b^	0.039
Muslim diet	28.2	20.0^a^	27.9^a,b^	37.2^b^	0.003	23.5^a,b^	19.9^b^	32.1^a,c^	43.8^c^	0.001
Energy-dense diet	59.7	42.5^a^	57.5^b^	80.8^c^	< 0.001	44.3^a^	53.6^a,b^	66.5^b,c^	78.1^c^	< 0.001
Fingerfood	54.5	38.8^a^	54.3^b^	71.2^c^	< 0.001	44.3^a^	46.4^a^	57.4^a^	80.8^b^	< 0.001
Recipes										
Available for most/all dishes	58.1	53.1^a^	53.8^a^	69.9^b^	0.002	53.9^a,b^	50.0^b^	63.2^a^	68.5^a^	0.007
*missing*	2.0	1.3	2.4	1.9		1.7	1.2	2.4	2.7	
Calculation of the energy and nutrient content available	66.6	67.5	62.3	72.4	0.383	68.7	65.1	62.2	79.5	0.125
*missing*	6.7	6.9	8.9	3.2		7.8	4.8	9.1	2.7	
Nutritional care										
Malnutrition screening										
Malnutrition screening at least 4–6 times a year	78.7	76.9	77.3	82.7	0.792	78.3	78.3	79.4	78.1	0.941
*missing*	6.2	6.9	7.7	3.2		7.8	5.4	6.2	5.5	
Dietetic counseling service	74.2	66.3^a^	72.1^a^	85.9^b^	< 0.001	65.2^a^	70.5^a^	77.0^a^	89.0^b^	< 0.001
*missing*	0.9	0.6	0.4	1.9		1.7	0.0	0.0	4.1	
Routine assessment of personal nutrition history	67.5	61.3	66.8	75.0	0.068	58.3^a^	66.3^a,b^	68.9^a,b^	80.8^b^	0.041
*missing*	4.3	6.9	3.2	3.2		6.1	6.0	2.9	1.4	
Management and quality assurance										
Staffing aspects										
Dietician available	41.9	33.1^a^	39.3^a^	55.1^b^	0.001	38.8	41.6	39.7	54.8	0.071
*missing*	5.2	5.0	6.1	3.8		6.1	4.8	4.3	6.8	
Interdisciplinary nutrition team	11.5	12.5	8.9	14.7	0.184	7.0^a^	8.4^a^	12.4^a,b^	23.3^b^	0.003
Quality circle for nutritional care	58.6	43.8^a^	63.2^b^	66.7^b^	< 0.001	50.4^a^	54.2^a,b^	62.2^a,b^	71.2^b^	0.015
Regular staff training	82.1	73.1^a^	83.0^a,b^	89.7^b^	0.001	74.8^a^	79.5^a^	83.3^a^	95.9^b^	0.002
Framework regulations										
Process instructions available	63.6	53.1^a^	64.0^a,b^	73.7^b^	0.001	47.8^a^	58.4^a,b^	72.2^c^	75.3^b,c^	< 0.001
Interface descriptions available	33.2	27.5	34.8	36.5	0.181	27.8^a^	30.1^a,b^	33.5^a,b^	47.9^b^	0.025
Regular resident satisfaction surveys	71.4	65.6	72.5	75.6	0.127	68.7	70.5	72.2	75.3	0.775

Differences by number of TM-levels and number of best practices for TMD were tested with Chi^2^-test and post-hoc z-test, respectively. Each subscript letter denotes a subset of “number of TM-levels” or “number of best practices for TMD” categories whose column proportions do not differ significantly from each other (*p* < 0.05)

#### Nutritional care

All aspects regarding nutritional care were implemented by at least two thirds of the NHs (Table 3). A dietetic counseling service was more frequently offered in NHs preparing three compared to NHs preparing one or two TM-levels (*p* < 0.001). Likewise, it was more frequently available in NHs considering four best practices for TMD compared to NHs considering one to three (*p* < 0.001). The routine assessment of the personal nutrition history was unrelated to the number of TM-levels, but was more frequent in NHs with four instead of NHs with only one best practices for TMD (80.0% vs. 58.3%, *p* = 0.041). No differences were observed concerning periodic malnutrition screening, with regard to both TMD aspects (Table 3).

#### Management and quality assurance

About 42% of the NHs worked with a dietician. The availability of a dietician was more common in NHs offering three instead of one or two TM-levels (*p* = 0.001) and in NHs offering four instead of one best practices for TMD (*p* = 0.071) (Table 3). An interdisciplinary nutrition team was established more frequently in NHs offering four compared to one or two best practices for TMD (*p* = 0.003). The likelihood for the presence of a quality circle for nutritional care and regular staff training increased with the number of offered TM-levels (*p* < 0.001, *p* = 0.001, respectively) and best practices for TMD (*p* = 0.015, *p* = 0.002, respectively) (Table 3).

Process instructions were more common in NHs offering three TM-levels (*p* = 0.001) or using four best practices for TMD (*p* < 0.001) than in NHs with one, respectively. Interface descriptions were available more often in NHs offering four instead of one best practices for TMD (*p* = 0.025). The implementation of regular surveys on resident satisfaction was not associated with either of the two TMD aspects (Table 3).

### Resident-related NH characteristics and TMD

The number of residents with swallowing disorders was reported by 478 NHs (84.9%), the number of residents with chewing disorders by 446 NHs (79.2%), and the number of residents receiving TMD by 513 NHs (91.1%).

Swallowing disorders had a median prevalence of 8.0% (IQR 4.0–13.5%, mean ± SD 10.5 ± 9.7%), with 22 NHs (4.6%) reporting zero residents with swallowing disorders. The median prevalence of chewing disorders was 8.8% (3.3–16.9%, 12.2 ± 12.5%), with 65 NHs (14.6%) stating having no residents with chewing disorders. On average, 14.3% (9.7–20.9%, 16.9 ± 11.2%) of all residents received TMD, with only three NHs (0.6%) reporting having no residents receiving TMD. No differences in these characteristics were revealed regarding the number of available TM-levels or best practices for TMD, except a higher median percentage of residents receiving TMD in NHs with three (16.3%) compared to in NHs with one (13.2%) available TM-level (*p* = 0.037).

## Discussion

To our knowledge, this is the first study describing the availability of TMD and the consideration of best practices for TMD in a large sample of German NHs. The study sample includes about 5% of all German NHs. Representativeness of our study sample is given regarding the regional distribution of the NHs and the proportion of non-profit institutions [16]. However, small NHs are underrepresented (17% vs. 30%) and medium-sized NHs slightly overrepresented (53% vs. 44%) compared to the actual situation in Germany (Federal Office of Statistics, www.gbe-bund.de). Unfortunately, no other information on nutrition-related nursing home characteristics is available on the national level for further comparison. Selective participation of institutions with specific interest in nutrition is very probable and must be taken into account.

Although all participating NHs offer some kind of TMD, there is obviously room for improvement, as only 7.6% of the institutions had three TM-levels available as well as four best practices for TMD implemented. Associations between several operational NH characteristics and compliance with these recommendations were found. Interestingly, the knowledge of the recommendations did not influence the implementation of TM-levels or best practices for TMD. No associations were found regarding structural and resident-related NH characteristics, except a slightly higher percentage of residents receiving TMD in NHs offering three TM-levels instead of one.

### Texture modified diet

In almost all NHs “minced and moist” texture is available, which is easy to produce as only a blender and no special knowledge is needed. Soft texture, offered by only 60% of the NHs, requires no special processing, but only a special range of products can be used that is naturally soft or become soft (fork-mashable) by cooking. The production of pureed texture is most elaborate as it should be lump free and requires special equipment (e.g. a bowl cutter) for several types of food (e.g. meat) because of the natural fiber content. The different requirements on time and equipment and insufficient knowledge – e.g. about the required consistency of each TM-level, and about the food and processing methods required to achieve the right texture – might explain their varying availability. Additionally, the necessity to offer different TM-levels to be able to meet residents’ individual needs might not be present. Furthermore, it is possible that some institutions did not state to offer a specific texture because none of the residents had the respective needs at that moment although the questionnaire asked for general availability of TM-levels.

Differing terminology, definitions and levels of TMD are described in the literature of individual countries, and there are even differences within small geographical regions within countries [6, 17]. Therefore, it is unclear whether the definitions used in the questionnaire were understood in the same way by all NHs although a short explanation was given.

Residents with swallowing and/or chewing disorders need TMD for a safe and adequate energy and nutrient intake. Texture- modified meals often look less appealing, taste different and can also have psychological impacts, e.g. embarrassment which can affect acceptance of TMD and quality of life. Therefore, it is important to consider best practices for TMD in addition to the availability of different TM-levels [2, 7, 18, 19]. The basic recommendation is to serve the meal with separately visible components that allow the residents to recognize the food and to eat and taste it as they are used to with a regular diet. Re-shaping the components enhances the visual appeal of the meals significantly. In an intervention study conducted in a long-term care facility in Canada, increased body weight, energy and nutrient intake were observed after 12 weeks in residents receiving re-shaped TMD compared to a control group receiving unshaped TMD [7]. Another study in the USA showed an increase in food intake by 15% after changing to 3-D preparation of pureed foods [19]. Missing knowledge, equipment, and especially time and financial resources but also lacking awareness of the importance of this aspect might be reasons for not re-shaping or separately presenting the meal components.

The recommendation to prepare texture-modified meals similar to regular meals should probably ensure that residents have the same choices and menu plans as residents on a regular diet. NHs with in-house meal production tend to follow this recommendation more frequently than NHs with external production (70% vs. 59%, *p* = 0.051). Not following this recommendation might also result from the use of commercially produced texture-modified foods.

The consideration of residents’ individual capabilities is important for menu planning in order to be able to offer residents meals that they are able to eat safely and at the same time process these meals as little as possible to minimize the impairment of the quality of life due to texture-modification [20]. At least one study indicated that the majority of NH residents (91%) received overly restrictive TMD [20]. One in five NHs that stated considering residents’ individual capabilities offered only one TM-level (Table 1). Therefore, it is unclear how that aspect was understood and implemented.

The positive association between availability of TM-levels and best practices for TMD indicates that NHs with a stronger focus on TMD considered both aspects simultaneously.

### Structural NH characteristics and TMD

Structural NH characteristics are given facts that are not related to individual people and cannot be changed easily or in the foreseeable future. The structural characteristics examined here, do not seem to influence the implementation of TMD recommendations. Solely in-house meal production tended to be more common in NHs considering four instead of one best practices for TMD (Table 2). This tendency might be explained by the direct contact of staff and residents to the kitchen, allowing for immediate feedback and the possibility to change preparation processes on short notice.

Associations between NH size, as one of the structural NH characteristics, and single best practices for TMD, but no TM-level, were shown in a previous publication [15]. Components were found to be re-shaped more frequently in large than in small NHs, and individual capabilities were more frequently considered in small than in large NHs [15]. The size and the affiliated resources of the NH seem to influence the implementation of single best practices for TMD contrariwise. This might be one reason why the size of the NH was not associated to the number of implemented best practices for TMD.

### Operational NH characteristics and TMD

Operational NH characteristics describe processes and the availability of special offers. Their implementation is usually dependent on a few persons in the NH and can be changed in the foreseeable future. The analyzed characteristics focus on nutrition aspects and their implementation may reflect a general nutritional awareness.

The majority of operational NH characteristics studied (13 out of 16) were associated either with the number of TM-levels or the number of implemented best practices for TMD, mostly with both (Table 3).

The availability of the analyzed special diets and delivery forms are also part of the national recommendations for nutrition in nursing homes [12]. This might be one reason for the positive association of them being offered and TMD. NHs following the recommendations for nutrition in NHs of the German Nutrition Society intentionally will probably consider all recommended aspects regarding special diets including TMD.

The calculation of energy and nutrient content is important as texture modification can lead to a dilution. A significantly lower mean energy provision of TMD compared to regular diet was shown in previous studies [21, 22]. NHs that do not calculate energy and nutrient content of TMD might not be aware of that and are at higher risk to provide insufficient amounts of energy and nutrients. The basis for calculating energy and nutrient content are written recipes, which also ensure a standardized preparation of meals. Those recipes are missing in 40% of the NHs. It is questionable how precise the calculation is made in some institutions as 32.9% of the 368 NHs that reported calculating energy and nutrient content do not have recipes for most/all dishes.

Dieticians are the food and nutrition experts in the institutions, but their roles can vary considerably [23]. Dieticians are, even in the group of German NHs offering three TM-levels (55%), clearly less common compared to NHs in other countries like the Netherlands (92%), Austria (84%) [24] and Italy (88%) [25].

A dietetic counseling service was common in participating NHs, however only half of the NHs (52%) offering the dietetic counseling service had a dietician available. Therefore, further information about the contents of the service and the qualification of the counselor would be of interest. The assessment of residents’ personal nutrition history is common as well, especially in NHs offering three texture-modified levels or four best practices for TMD. Although it is a good way to improve the quality of life by offering meals that meets the residents’ wishes, it is unclear how the collected information is used in daily practice.

The implementation of periodic malnutrition screening that was recommended and controlled by the medical service of health insurances in Germany, an institution controlling nursing care routinely, was not associated with the implementation of TMD. Interestingly, in spite of annual controls, 15% of the NHs do not periodically screen for malnutrition.

The assessed management and quality assurance aspects describe tools and processes to improve special knowledge, teamwork and communication. Except for the regular resident satisfaction survey, all aspects are associated with the offer of TMD (Table 3), leading to the assumption that an interdisciplinary approach with established processes and lines of communication influences the offer of TMD, probably in different ways. One aspect might be the general nutritional awareness that initially promotes the establishment of these management and quality assurance aspects. Additionally, different views and feedback resulting from these structures might directly influence the offer of TMD.

Regular resident satisfaction surveys that are assumed to influence meal quality through direct feedback were not associated with both TMD aspects. Reasons might be that TMD is not part of these surveys, that residents on TMD cannot take part because of cognitive or physical impairment, or that the results did not induce any changes in TMD.

### Resident-related NH characteristics and TMD

Swallowing and chewing problems are the main reasons for the implementation of TMD [26–28]. Cognitive deficits, refusal to eat, chew or swallow, and efficiency in feeding were other common reasons reported in combination with swallowing or chewing problems [26, 27]. The prevalence rates reported by the NHs varied widely. The median prevalence of swallowing problems (8%) was slightly below the results of two large studies assessing the prevalence at resident level, the LPZ study in the Netherlands including 3220 NH residents (11%) [29] and the worldwide nutritionDay study including 23,549 NH residents (13%) [30]. Other studies using specific dysphagia screening tests instead of asking staff or residents, reported markedly higher prevalence numbers (38 to 70%) [31–33]. The median prevalence of chewing problems (9%) was lower compared to 26% in the LPZ study and the nutritionDay study [29, 30] and is probably underestimated by those completing our questionnaire.

Information on the frequency of TMD use is rare. In our study, the median percentage of residents receiving TMD was 14% (range 0–83%). In a study conducted in the USA in 1989, 292 long-term care institutions asked for the percentage of residents receiving TMD reported an average of 42% [27]. Other studies also reported a more frequent use of TMD with 26% [26], 29% [8] and 67% [22] of the residents receiving this diet, but these studies were limited to only one or two NHs. In NHs participating in the nutritionDay project, 13% of the residents received TMD which is similar to our result [30].

In NHs offering three TM-levels the proportion of residents receiving TMD was significantly higher than in NHs with one TM-level, due to a few institutions with more than 40% of residents receiving TMD. Regardless of the number of TM-levels, about half of the NHs reported between 10 and 20% of their residents being on TMD. Nevertheless, there are NHs with one out of five residents being on TMD offering only one TM-level. Even if the proportion is low, individuals might need a specific TM-level. Since we do not have detailed information on the severity of swallowing or chewing problems and the TM-level received, an evaluation of the quality of their nutritional care is not possible.

### Strengths and limitations

The strength of our study is the large number of assessed aspects, which allows good characterization of the NHs on institutional level. Additionally, the distribution of existing and participating NHs throughout Germany is comparable. The sample size is large compared to the only other study found with focus on TMD on institutional level including 292 institutions [27]. Other studies, reporting about prevalence of TMD on resident level, were limited to one or two institutions [8, 22, 26]. Nevertheless, the response rate is low (7.2%).

The length of the questionnaire might have limited motivation to participate. The accuracy of completion (e.g. regarding the estimation of the proportion of residents receiving TMD) was dependent on the knowledge and motivation of one or two people of the NH. Another constraint of the study is the limited information that can be assessed by a survey. The data collection with the help of a questionnaire might have led to more socially desirable than true answers.

The voluntary participation in the study presumably led to a bias of NHs with special interest in nutrition and comprehensive data on German NHs for a comparison is missing. Furthermore, the information on resident characteristics was limited to 79–91% of the participating NHs.

## Conclusion

Although all participating NHs offer some kind of TMD, improvement in range and preparation of TMD is necessary as only 7.6% of the NHs meet all recommendations regarding TMD and best practices for TMD. The implementation of these recommendations is important, on the one hand to allow NH residents to eat at all, to ensure their safety during the swallowing process and to support them in retaining their ability to eat, and on the other hand to increase their quality of life, their satisfaction and to maintain their nutritional status when receiving TMD.

This study identifies several operational NH characteristics with strong associations to TMD and best practices for TMD. These characteristics reflect a general nutritional awareness. As operational aspects are usually dependent on a few persons in the institution and are changeable in the foreseeable future, strategies should be developed to address these key-persons and to spark their interest in nutrition, especially under consideration that knowledge of national recommendations for TMD did not influence the implementation of TMD and best practices for TMD.

Future research is needed to identify the reasons for not following the recommendations for TMD in order to develop an approach for a wider implementation. Furthermore, the relationship between TMD and operational NH characteristics should be analyzed in more detail to identify the cause of the correlations. In addition, the quality regarding energy and nutrient content of TMD and whether the residents receive the TM-level they actually need should be assessed.

## Supplementary information

**Additional file 1:** Questionnaire that was used for the survey. (DOCX 84 kb)

**Additional file 2:** Table containing the availability of the different combinations of TM-levels. (DOCX 12 kb)

**Additional file 3:** Table containing the availability of the different combinations of best practices for TMD. (DOCX 12 kb)

**Additional file 4:** Table showing the knowledge of national recommendations for nutrition overall and according to the number of TM-levels and best practices for TMD. (DOCX 13 kb)

## Data Availability

The authors declare that the data supporting the findings of this study are available within the article and the supplementary information files.

## Abbreviations

DGE: German Nutrition Society
NHs: nursing homes
TMD: texture-modified diet
