# Supplementary material for: Texture modified diet in German nursing homes: availability, best practices and association with nursing home characteristics
Source: BMC Geriatr. 2019 Oct 23;19:284. doi: 10.1186/s12877-019-1286-9 (PMC6806511; doi:10.1186/s12877-019-1286-9)
Supplement: Supplementary file 1 — Additional file 1: Questionnaire that was used for the survey. (DOCX 84 kb) [file 12877_2019_1286_MOESM1_ESM.docx]

**Research study on nutrition in nursing homes**

| **Structural characteristics** |
| --- |

| 1. **In which federal state is the nursing home located?** | | | |  |
| --- | --- | --- | --- | --- |
| 🞎 Baden-Württemberg | 🞎 Bremen | 🞎 Lower Saxony | 🞎 Saxony | |
| 🞎 Bavaria | 🞎 Hamburg | 🞎 North Rhine-Westphalia | 🞎 Saxony-Anhalt | |
| 🞎 Berlin | 🞎 Hesse | 🞎 Rhineland-Palatinate | 🞎 Schleswig-Holstein | |
| 🞎 Brandenburg | 🞎 Mecklenburg-West   Pomeria | 🞎 Saarland | 🞎 Thuringia | |

| 1. **The institution is located in a city with the following number of inhabitants:** | | | | |
| --- | --- | --- | --- | --- |
| 🞎 < 5,000 | 🞎 5,000 – 20,000 | 🞎 > 20,000 – 100,000 | 🞎 > 100,000 – 500,000 | 🞎 > 500,000 |

| 1. **Who is the responsible body for the institution?** | | | |
| --- | --- | --- | --- |
| 🞎 Non-profit (e.g. AWO, Caritas, etc.) | 🞎 Private | 🞎 Government-owned (e.g. the city) |  |

| 1. **How big is the institution?** Number of beds: long-term care ____ short term care ____ day care ___ |
| --- |

| 1. **Which housing concept is established in the institution?^[[1]](#footnote-1)^** | |
| --- | --- |
| 🞎 „Stationskonzept“ | 🞎 „Hausgemeinschaftskonzept/Alltagsweltorientierte Wohngruppen“ |
| 🞎 „Wohnbereichskonzept“ | 🞎 others _______________________________________ |

| 1. **Does the institution have any certificates? (e.g. DIN ISO 9001, EFQM)?** 🞎 yes 🞎 no | |
| --- | --- |
| If so, which certificates? | ________________________________________________ |

| 1. **Resident-related characteristics:** |
| --- |
| How many residents are in each care level? *(please state numbers)* |

| No care level _____ | Care level 1 _____ | Care level 2 _____ | Care level 3 _____ |
| --- | --- | --- | --- |

| How many residents have dementia? | ____________ residents |
| --- | --- |
| How many residents have swallowing disorders? | ____________ residents |
| How many residents have chewing disorders? | ____________ residents |

| How many residents are overweight (BMI higher than 30)? | ____________ residents |
| --- | --- |
| How many residents are underweight (BMI less than 20)? | ____________ residents |

| How many residents are tube fed? | ____________ residents |
| --- | --- |

| 1. **Which professional qualification has the head of the kitchen in the institution?** |  |
| --- | --- |
| ______________________________________________________________________________________ | |

| 1. **Is a person specialized in nutrition (e.g. a dietician) available in the institution?** 🞎 yes 🞎 no |
| --- |

| **Food provision** |
| --- |

| 1. **Is catering part of the guiding principle of the institution?** 🞎 yes 🞎 no |
| --- |

| 1. **The hot meals are…** | 🞎 produced in the institution. |
| --- | --- |
|  | 🞎 delivered (hot or for regeneration). |

If delivered: Is the caterer certified according to DGE recommendations?^[[2]](#footnote-2)^ 🞎 yes 🞎 no

| 1. **Which system is used for hot meal production?** | |
| --- | --- |
| 🞎 Cook & Serve *(prepared on site)* | 🞎 Cook & Hold *(delivered hot))* |
| 🞎 Cook & Chill *(delivered chilled for regeneration)* | 🞎 Cook & Freeze *(delivered deep frozen for regeneration)* |

| 1. **Who is the operator of the kitchen?** | | |
| --- | --- | --- |
| 🞎 Owner-operated | 🞎 Service company of the responsible body | 🞎 Caterer |

| 1. **Does the kitchen provide meals additionally for external catering participants?** 🞎 yes 🞎no | | | | |  |
| --- | --- | --- | --- | --- | --- |
| If so, please add the average number of daily meals. | | | | | |
| Open lunch/day care | ______ | School ______ | Meals on wheels ______ | | |
| Other nursing homes | | ______ | Nursery ______ | Others _____________ ______ | |

| 1. **How often are meals or meal components produced locally at ward level?** | | | | | |  |
| --- | --- | --- | --- | --- | --- | --- |
| Hot meals | 🞎 several times per day | 🞎 once daily | 🞎 several times per week | 🞎 rarely | 🞎 never | |
| Cold meals | 🞎 several times per day | 🞎 once daily | 🞎 several times per week | 🞎 rarely | 🞎 never | |

| 1. **Are snacks available at any time?** 🞎 yes 🞎 no |
| --- |

| 1. **Do you offer „eat by walking“ in the institution?** 🞎 yes 🞎 no |
| --- |

| 1. **Which distribution system is used for lunch?** | dining room/restaurant | | ward | room |  |
| --- | --- | --- | --- | --- | --- |
| Tray food | | 🞎 | 🞎 | 🞎 | |
| Bulk food | | 🞎 | 🞎 | 🞎 | |
| Family style meals | | 🞎 | 🞎 |  | |
| Buffet | | 🞎 | 🞎 |  | |
| Pre-plated meals | | 🞎 | 🞎 | 🞎 | |

| 1. **Is it possible to switch time of meals?** 🞎 yes, all meals 🞎 yes, cold meals 🞎 no |
| --- |

| 1. **During which time periods are the main meals available for the residents?** | | | |
| --- | --- | --- | --- |
| breakfast | from __________ | until __________ o‘clock |  |
| lunch | from __________ | until __________ o‘clock |  |
| dinner | from __________ | until __________ o‘clock |  |

| 1. **Which service features and assistance do the residents receive?** *(multiple responses possible)* | |
| --- | --- |
| 🞎 counseling and help with food choice | 🞎 naming of meals and components at provision |
| 🞎 request of residents‘ wishes and portion sizes | 🞎 dietetic counseling service |
| 🞎 support with eating and drinking | 🞎 provision of special feeding devices (e.g. special cutlery) |

1. **Which budget (net) is available for the kitchen per day and per resident for cost of goods?** ________ €

| **Food selection** |
| --- |

| 1. **Which beverages are available for the residents at any time?** *(multiple responses possible)* | | | | | |
| --- | --- | --- | --- | --- | --- |
| 🞎 tea | 🞎 coffee | 🞎 mineral water | 🞎 table water | 🞎 juices(100% fruit) | 🞎 others _________ |

| 1. **How many portions of the following food groups are offered on average in one menu line (regular diet) on one day (full board)?** | | | | | | |
| --- | --- | --- | --- | --- | --- | --- |
| Bread, potatoes, rice, pasta and other cereal products | 🞎 more than 3 | 🞎 3 | 🞎 2 | 🞎 1 | 🞎 less than 1 portion/day | |
| Whole-grain products (e.g. oatmeal, bread, cake, etc.) | 🞎 more than 3 | 🞎 3 | 🞎 2 | 🞎 1 | 🞎 less than 1 portion/day | |
| Vegetables (fresh or frozen), salad,  legumes (e.g. beans, lentils) | 🞎 more than 3 | 🞎 3 | 🞎 2 | 🞎 1 | 🞎 less than 1 portion/day | |
| Fruit (fresh or frozen, as well as stewed or as part of desert) | 🞎 more than 3 | 🞎 3 | 🞎 2 | 🞎 1 | 🞎 less than 1 portion/day | |
| Milk, cheese, yoghurt, curd and deserts made of these | 🞎 more than 3 | 🞎 3 | 🞎 2 | 🞎 1 | 🞎 less than 1 portion/day | |

| 1. **How often do you offer the following whole-grain products during one week?** | | | | | | |
| --- | --- | --- | --- | --- | --- | --- |
| Whole-grain bread | 🞎 daily | 🞎 3-4x/week | 🞎 1-2x/ week | 🞎 rarely | 🞎 never |  |
| Whole-grain semolina | 🞎 daily | 🞎 3-4x/ week | 🞎 1-2x/ week | 🞎 rarely | 🞎 never |  |
| Whole-grain pasta | 🞎 daily | 🞎 3-4x/ week | 🞎 1-2x/ week | 🞎 rarely | 🞎 never |  |
| Brown rice | 🞎 daily | 🞎 3-4x/ week | 🞎 1-2x/ week | 🞎 rarely | 🞎 never |  |

| 1. **a. How often do you offer fish (e.g. fish fillet, herring salad) in one menu line in one week (full board)   on average?** | | | | | | | | | |
| --- | --- | --- | --- | --- | --- | --- | --- | --- | --- |
| 🞎 more than 3 | | 🞎 3 | 🞎 2 | | 🞎 1 | | 🞎 less than 1x/week | |  |
| **b. Do you use fish/-products from sustainable fishing, e.g. MSC-labeled fish?** | | | | | | | | |  |
| 🞎 always | 🞎 partly | | | 🞎 never | | 🞎 unknown | |  |  |

| 1. **How often do you offer meat, meat products or sausages in one menu line in one week (full board) in average?** | | | | | | | |
| --- | --- | --- | --- | --- | --- | --- | --- |
| 🞎 more than 5 | 🞎 5 | 🞎 4 | 🞎 3 | 🞎 2 | 🞎 1 | 🞎 less than 1x/week |  |

| 1. **Do you or does your caterer use rape oil as standard oil?** 🞎 yes 🞎 no 🞎 unknown |
| --- |

| **Menu planning** |
| --- |

| 1. **How long is the cycle for menu planning for lunch before repetition?** | __________ weeks |
| --- | --- |

| 1. **How many menu lines do you offer for lunch?** | __________ menu lines |
| --- | --- |

| 1. **Do residents have free choice of side dishes at lunch?** 🞎 yes 🞎 no |
| --- |

| 1. **Which diets are available?** *(multiple responses possible)* | | | | |
| --- | --- | --- | --- | --- |
| 🞎 regular | 🞎 light-regular | 🞎 vegetarian | 🞎 diabetes | 🞎 lower cholestrol |
| 🞎 energy-dense | 🞎 energy-restricted | 🞎 low in sodium | 🞎 low in purine | 🞎 gluten-free |
| 🞎 lactose-free | 🞎 preferenced-based | 🞎 fingerfood | 🞎 muslim | 🞎 _____________ |

| 1. **How many residents are on regular or light-regular diet?** | __________ residents |
| --- | --- |

| 1. **Which levels of texture-modified diet are provided?** *(multiple responses possible)* | |
| --- | --- |
| 🞎 soft texture *(not pureed)* | 🞎 pureed *(strained through a sieve)* |
| 🞎 „minced and moist“ *(finely minced)* | 🞎 „smoothfood“ |

| 1. **Which of the following best practices are considered for preparation of texture-modified diet?** *(multiple responses possible)* | | |
| --- | --- | --- |
| 🞎 components are seperately visible | 🞎 components are re-shaped |  |
| 🞎 components are derived from menu plan | 🞎 individual capabilities of the residents are considered |  |

| 1. **How many residents receive texture-modified diet?** | __________ residents |
| --- | --- |

| 1. **a. Are written recipes available for meal preparation?** | | | |
| --- | --- | --- | --- |
| 🞎 yes, for all dishes | 🞎 yes, for most dishes | 🞎 yes, for some dishes | 🞎 no |
| **b. Do the recipes contain explicit information regarding preparation?** | | | |
| 🞎 yes, for all recipes | 🞎 yes, for most recipes | 🞎 yes, for some recipes | 🞎 no |

| 1. **Do you calculate the energy and nutrient content of at least one menu line (full board)?** 🞎 yes 🞎 no |  |
| --- | --- |
| If so, does the energy and nutrient content comply with DGE recommendations?  🞎 yes 🞎 no 🞎 unknown | |
| Do you use software for the calculation of the energy and nutrient content? 🞎 yes 🞎 no | |

| 1. **How are residents’ wishes for menu planning assessed?** *(multiple responses possible)* | |
| --- | --- |
| 🞎 assessment of nutritional history | 🞎 interview with residents by care staff |
| 🞎 posting of a wishlist | 🞎 interview with residents by the head of the kitchen |
| 🞎 interview with relatives | 🞎 interview with residents by service staff |
| 🞎 others ______________________________________________________________________ | |

| **Management, quality assurance and nutritional care** |
| --- |

| 1. **How often do you screen residents for malnutrition/malnutrition risk?** | | | | | | | |  |
| --- | --- | --- | --- | --- | --- | --- | --- | --- |
| 🞎 once at admission | | 🞎 1-2 times/year | | 🞎 4-6 times/year | | 🞎 about once in a month | 🞎 never | |
| If you screen, which tool do you use? | | | | | | | | |
| 🞎 MNA – long form | 🞎 MNA – short form | | 🞎 PEMU | | 🞎 others ____________________________ | | | |

| 1. **Which nutritional interventions are used for residents with malnutrition or high risk of malnutrition?** | | |
| --- | --- | --- |
| 🞎 „rich“ meals *(e.g. added cream, special shakes)* | 🞎 nutrient concentrates *(e.g. maltodextrin)* | |
| 🞎 balanced supplement powder | 🞎 balanced sip feed | |

| 1. **Is a nutritional history of each resident available?** 🞎 yes 🞎 no |
| --- |

| 1. **What kind of quality assurance is established in the institution?** *(multiple responses possible)* | |  |
| --- | --- | --- |
| 🞎 complaint management | 🞎 resident satisfaction surverys | |
| 🞎 interviews with relatives | 🞎 assessment of preferences and aversions of the residents | |
| 🞎 regular quality audits | 🞎 coordination with resident representatives | |
| 🞎 staff training | 🞎 to date, none | |
| 🞎 others _______________________________________________________________________ | | |

| 1. **Which aspects are part of the written catering concept in the institution?** *(multiple responses possible)* | |
| --- | --- |
| 🞎 special diets | 🞎 offers for residents with special needs (e.g. dementia) |
| 🞎 meal times | 🞎 management of malnutrition |
| 🞎 interface regulations | 🞎 „end of life“ nutrition |
| 🞎 mealtime environment | 🞎 no written catering concept available |

| 1. **How is the interface management between kitchen, housekeeping and care staff organized in the institution?** *(multiple responses possible)* | |
| --- | --- |
| 🞎 assignments of tasks and functions | 🞎 regular quality circle of nutritional care |
| 🞎 interface descriptions | 🞎 interface manager |
| 🞎 process instructions | 🞎 nutrition representative |
| 🞎 interdisciplinary nutrition team | 🞎 no special regulations |
| 🞎 others _______________________________________________________________________ | |

| 1. **Is a HACCP-concept available in the institution?** 🞎 yes 🞎 no |
| --- |

| 1. **Which strategies are used to ensure good hygiene?** *(multiple responses possible)* | |
| --- | --- |
| 🞎 regular training | 🞎 temperature measurements (e.g. during preparation, meal provision) |
| 🞎 cleaning and hygiene plan | 🞎 process instructions for best practice (e.g. hand hygiene) |
| 🞎 reference samples | 🞎 others ________________________________________________ |

| 1. **a. Do you know the DGE recommendations for nutrition in nursing homes?** 🞎 yes 🞎 no *(end of survey)* | | | | | | | | | | | |
| --- | --- | --- | --- | --- | --- | --- | --- | --- | --- | --- | --- |
| **b. How did you take notice of the DGE recommendations?** *(multiple responses possible)* | | | | | | | | | | | |
| 🞎 internet | | 🞎 DGE (e.g. homepage) | | | | 🞎 journal | | | | 🞎 brochure | |
| 🞎 trade fair | | 🞎 education and training | | | | 🞎 colleague | | | | 🞎 others ___________ | |
| **c. Are the DGE recommendations helpful for you?** 🞎 yes 🞎 no | | | | | | | | | | | |
| If so, which of the topics of the recommendations have helped you? *(multiple responses possible)* | | | | | | | | | | | |
| 🞎 selcetion of foods | | | | 🞎 menu planning | | | | | 🞎 legal regulations | | |
| 🞎 preparation of meals | | | | 🞎 quality assurance | | | | | 🞎 dining atmosphere | | |
| 🞎 malnutrition | | | | 🞎 special diets | | | | | 🞎 others _____________________ | | |
| **d. Do you follow the DGE recommendations in your institution?** | | | | | | | | | | | |
| 🞎 yes | 🞎 partly | | | | 🞎 no | | | | | | |
| **e. Why do you not or just partly follow the DGE recommendations?** *(multiple responses possible)* | | | | | | | | | | | |
| 🞎 costs/financial resources | | | | | | | 🞎 scarce time resources | | | | |
| 🞎 insufficient staff qualification | | | | | | | 🞎 inadequate kitchen equipement | | | | |
| 🞎 difficult to integrate in daily practice | | | | | | | 🞎 do not meet residents needs | | | | |
| 🞎 lack of acceptance of whole grain products | | | | | | | 🞎 lack of acceptance of fruit and vegetables | | | | |
| 🞎 lack of acceptance of vegetarian meals | | | | | | | 🞎 others _________________________________ | | | | |
| **f. Do you consider a certification according to the DGE recommendations?** | | | | | | | | | | | |
| 🞎 yes, already certified | | | 🞎 yes, in process | | | | | 🞎 yes, in planning | | | 🞎 no |

**Thank you for participation!**

Please attach menu plans for a period of 6 weeks. All information will be analyzed anonymized.

In the following part you may provide us with additional feedback.

**Your wishes and suggestions regarding the DGE recommendations**

________________________________________________________________________________________________________

________________________________________________________________________________________________________

________________________________________________________________________________________________________

________________________________________________________________________________________________________

________________________________________________________________________________________________________

**What are the biggest challenges in your daily catering routine?**

________________________________________________________________________________________________________

________________________________________________________________________________________________________

________________________________________________________________________________________________________

________________________________________________________________________________________________________

________________________________________________________________________________________________________

| **What is special with the catering in your institution?** |
| --- |

________________________________________________________________________________________________________

________________________________________________________________________________________________________

________________________________________________________________________________________________________

________________________________________________________________________________________________________

________________________________________________________________________________________________________

| **What else do you want to tell us:** |
| --- |

________________________________________________________________________________________________________

________________________________________________________________________________________________________

________________________________________________________________________________________________________

________________________________________________________________________________________________________

________________________________________________________________________________________________________

**Thank you very much** – we will analyze the information anonymized.

1. The housing concepts are established by the German Society of the Aged (KDA - Kuratorium Deutsche Altershilfe). [↑](#footnote-ref-1)
2. DGE = German Nutrition Society [↑](#footnote-ref-2)
