# Supplementary material for: Texture modified diet in German nursing homes: availability, best practices and association with nursing home characteristics
Source: BMC Geriatr. 2019 Oct 23;19:284. doi: 10.1186/s12877-019-1286-9 (PMC6806511; doi:10.1186/s12877-019-1286-9)
Supplement: Supplementary file 2 — Additional file 2: Table containing the availability of the different combinations of TM-levels. (DOCX 12 kb) [file 12877_2019_1286_MOESM2_ESM.docx]

Supplemental material 1: Availability of the different combinations of TM-levels [%].

|  | **AvailableTM-levels** | **Total** (n=563) |
| --- | --- | --- |
| 1 TM-level | Soft | 0.7 |
|  | “minced and moist” | 25.2 |
|  | pureed | 2.5 |
| 2 TM-levels | Soft + “minced and moist” | 29.8 |
|  | Soft + pureed | 1.6 |
|  | Pureed + “minced and moist” | 12.4 |
| 3 TM-levels | Soft + “minced and moist” + pureed | 27.7 |
