# Supplementary material for: Texture modified diet in German nursing homes: availability, best practices and association with nursing home characteristics
Source: BMC Geriatr. 2019 Oct 23;19:284. doi: 10.1186/s12877-019-1286-9 (PMC6806511; doi:10.1186/s12877-019-1286-9)
Supplement: Supplementary file 3 — Additional file 3: Table containing the availability of the different combinations of best practices for TMD. (DOCX 12 kb) [file 12877_2019_1286_MOESM3_ESM.docx]

Supplemental material 2: Availability of the different combinations of best practices for TMD [%].

|  | **Considered** best practices for TMD | **Total** (n=563) |
| --- | --- | --- |
| 1 best practice for TMD | Components separately visible | 10.1 |
|  | Components similar to menu plan | 6.4 |
|  | Components re-shaped | 0.9 |
|  | Individual capabilities considered | 2.7 |
| 2 best practices for TMD | Components separately visible + Components similar to menu plan | 11.2 |
|  | Components separately visible + Components re-shaped | 3.0 |
|  | Components separately visible + Individual capabilities considered | 10.3 |
|  | Components similar to menu plan + Components re-shaped | 0.5 |
|  | Components similar to menu plan + Individual capabilities considered | 4.1 |
|  | Components re-shaped + Individual capabilities considered | 0.4 |
| 3 best practices for TMD | Components separately visible + Components similar to menu plan + Components re-shaped | 5.0 |
|  | Components separately visible + Components similar to menu plan + Individual capabilities considered | 27.0 |
|  | Components separately visible + Components re-shaped + Individual capabilities considered | 4.3 |
|  | Components similar to menu plan + Components re-shaped + Individual capabilities considered | 0.9 |
| 4 best practices for TMD | Components separately visible + Components similar to menu plan + Components re-shaped + Individual capabilities considered | 13.0 |
