# Supplementary material for: Texture modified diet in German nursing homes: availability, best practices and association with nursing home characteristics
Source: BMC Geriatr. 2019 Oct 23;19:284. doi: 10.1186/s12877-019-1286-9 (PMC6806511; doi:10.1186/s12877-019-1286-9)
Supplement: Supplementary file 4 — Additional file 4: Table showing the knowledge of national recommendations for nutrition overall and according to the number of TM-levels and best practices for TMD. (DOCX 13 kb) [file 12877_2019_1286_MOESM4_ESM.docx]

Supplemental material 3: Knowledge of national recommendations for nutrition in NHs (overall and according to number of TM-levels and best practices for TMD) [%].

|  |  | **Number of TM-levels** | | |  | **Number of** best practices for TMD | | | |  |
| --- | --- | --- | --- | --- | --- | --- | --- | --- | --- | --- |
|  | **Total** (n=563) | **1**  (n=160) | **2**  (n=247) | **3**  (n=156) | **p** | **1**  (n=115) | **2**  (n=166) | **3**  (n=209) | **4**  (n=73) | **p** |
| **National recommendations known** | 69.8 | 64.4 | 70.4 | 74.4 | 0.116 | 66.1 | 66.3 | 73.2 | 74.0 | 0.371 |
| *missing* | 1.4 | 1.3 | 1.2 | 1.9 |  | 1.7 | 1.8 | 1.0 | 1.4 |  |

Differences by number of TM-levels and number of best practices for TMD were tested with Chi^2^-test and post-hoc z-test, respectively.
